# Tearing down inequalities in the healthcare system across Europe: the BEACON project

**DOI:** 10.3389/fpubh.2025.1520772

**Published:** 2025-06-05

**Authors:** Chrysanthi Koukoutzeli, Giulia Ferraris, Veronica Coppini, Maria Vittoria Ferrari, Elisa Fragale, Dario Trapani, Ida Minchella, Roberto Grasso, Giuseppe Curigliano, Gabriella Pravettoni

**Affiliations:** ^1^Division of New Drugs and Early Drug Development for Innovative Therapies, European Institute of Oncology (IEO), IRCCS, Milan, Italy; ^2^Applied Research Division for Cognitive and Psychological Science, European Institute of Oncology (IEO), IRCCS, Milan, Italy; ^3^Department of Oncology and Hematology-Oncology, University of Milan, Milan, Italy

**Keywords:** cancer, health disparities, survival, new therapies, new treatment, time to reimbursement, Europe, socioeconomic status

## Abstract

Equity in healthcare remains a pressing issue in cancer care across the European Union. Although numerous European initiatives address prevention, early diagnosis, and treatment, significant disparities in access to innovative cancer therapies persist. Time-to-reimbursement for new anticancer drugs varies widely between member states, depending on national health policies, economic capacity, and healthcare infrastructure. These differences particularly affect countries in Central and Eastern Europe, where delays in reimbursement, limited access to clinical trials, and restricted availability of specialized care contribute to worse outcomes. This narrative review examines how disparities in reimbursement timelines and access to new cancer therapies may affect factors such as early detection, specialized treatment availability, clinical trial participation, and socioeconomic status. The discussion is framed within the BEACON project, a European Union-funded initiative under the EU4Health programme. BEACON brings together patients, healthcare providers, researchers, and policymakers to create a cross-border network for quality-assured diagnosis and treatment. Through its multilingual digital platform, the project fosters collaboration, supports health literacy, and enhances access to innovative cancer therapies, aiming to reduce inequities regardless of geographic or socioeconomic background.

## Introduction

“*There is nothing more unequal than the equal treatment of unequals*.” (Aristotle, *Nicomachean Ethics*) (1). This statement represents one of the earliest reflections on the principle of equity, a concept that remains profoundly relevant to this day, particularly in the context of cancer care across countries within the European Union.

The European Union (EU) identifies the reduction of health inequalities as a central policy objective. This commitment is evident across multiple initiatives, including those focused on prevention, early detection, treatment, and follow-up strategies, as reflected in numerous projects targeting cancer related health disparities (Table 1). Cancer survival rates are improving across European countries, despite an increased in incidence over the past decade, primarily driven by population aging and unhealthy lifestyle (2, 3). Encouragingly, predictions for cancer mortality in 2024 are favorable, attributed to advancements in early diagnosis and even more significantly, to improvements in treatment and disease management (3, 4).

**Table 1:** Several recent and ongoing European projects aimed at reducing cancer health inequalities.

Overview of European projects addressing cancer health inequities			
Project name	Description	Timeline	References
Europe’s Beating Cancer Plan (57)	Europe’s Beating Cancer Plan seeks to address the full spectrum of the disease. It focuses on four main areas where the EU can have the greatest impact: (1) prevention; (2) early detection; (3) diagnosis and treatment; and (4) improving the quality of life for cancer patients and survivors.	Ongoing (since 2020)	Europe’s Beating Cancer Plan Communication from the commission to the European Parliament and the Council.
ECIR (72) (European Cancer Inequalities Registry)	The European Cancer Inequalities Registry, a key initiative of Europe’s Beating Cancer Plan, offers accurate and dependable data on cancer prevention and care. It aims to highlight trends, disparities, and inequalities between different Member States and regions.	Launched in 2022	European Cancer Inequalities Registry (ECIR) | ECIR – European Cancer Inequalities Registry. https://cancer-inequalities.jrc.ec.europa.eu/.
JARC (73) (Joint Action on Cancer Control)	The funding mechanism established under the EU Health Programme aims to implement priority actions in the health sector at the EU level. Joint Actions are collaboratively coordinated by Member States or their designated bodies in partnership with the European Commission.	2017–2020	EU Joint Action on Rare Cancers (JARC): SIOP Europe. https://siope.eu/activities/eu-projects/eu-joint-action-rare-cancers-jarc/.
CanCon (74) (European Guide on Quality Improvement in Comprehensive Cancer Control)	This Joint Action had two primary objectives: (i) to identify essential components and quality standards for cancer control across Europe in order to mitigate disparities and inequalities; and (ii) to promote cooperation among Member States. This encompasses the sharing of best practices and the identification and definition of key elements necessary for delivering optimal and comprehensive cancer care.	2014–2017	Albreht, T., Kiasuwa, R. and Van Den Bulcke, M. European Guide on Quality Improvement in Comprehensive Cancer Control.
Can.Heal (75)	Focus on utilizing next-generation sequencing technology and identifying implementation pathways to enhance the application of genetic profiling for structured omics use in patient care. This approach aims to facilitate data sharing among EU Cancer Centres, improving equity in treatment and enabling more effective counseling regarding cancer risk through molecular tumor profiling biomarkers. They seek to establish a framework for integrating the Genome of Europe biobanking initiative into public health genomics for cancer.		Projects—EAPM. https://euapm.eu/projects/.
HTA4Patients (76) (Advanced the European Patients’ Academy on Therapeutic Innovation Training for Increased Patient Involvement in Health technology assessment)	Aims to integrate health technology assessments into patient pathways for better cancer care.	2021–2024	HTA4Patients—European Commission. https://health.ec.europa.eu/non-communicable-diseases/cancer/europes-beating-cancer-plan-eu4health-financed-projects/projects/hta4patients_en.
ORION (77) (Joint Action on Contribution to the Cancer Inequalities Registry to Monitor National Cancer Control Policies)	Focuses on improving the integration of new diagnostic and therapeutic technologies in cancer care.	2021–2024	OriON—European Commission. https://health.ec.europa.eu/non-communicable-diseases/cancer/europes-beating-cancer-plan-eu4health-financed-projects/projects/orion_en.
EUCAPA (78) (European Capacity Building for Patients)	Aims to improve cancer patient pathways and integrate care across different levels of health services.	2021–2024	EUCAPA—European Commission. https://health.ec.europa.eu/non-communicable-diseases/cancer/europes-beating-cancer-plan-eu4health-financed-projects/projects/eucapa_en.
Transition (79) (Digital Transition And Digital Resilience In Oncology)	Focuses on transitioning cancer patients from hospital to home care with enhanced support systems.	2021–2024	TRANSiTION—European Commission. https://health.ec.europa.eu/non-communicable-diseases/cancer/europes-beating-cancer-plan-eu4health-financed-projects/projects/transition_en.
Interact Europe 100 (80) (Implementing inter-specialty cancer training in 100 centres across Europe)	A project to enhance patient participation in decision-making processes for cancer treatment	2021–2024	IN INTERACT-EUROPE 100—European Commission. https://health.ec.europa.eu/non-communicable-diseases/cancer/europes-beating-cancer-plan-eu4health-financed-projects/projects/interact-europe-100_en.
DigiCanTrain (81) (Digital Skills Training for Health Care Professionals in Oncology)	Aims to develop digital training tools for healthcare professionals in cancer diagnosis and treatment.	2021–2024	DigiCanTrain—European Commission. https://health.ec.europa.eu/non-communicable-diseases/cancer/europes-beating-cancer-plan-eu4health-financed-projects/projects/digicantrain_en.
EU-CanIneq (82) (Supporting the Cancer Inequalities Registry to map disparities and inequalities between Member States and regions with a focus on socio-economic inequalities)	Registry, analyze data on total cancer mortality and mortality and produce country-specific information.	2021–2024	EU-CanIneq—European Commission. https://health.ec.europa.eu/non-communicable-diseases/cancer/europes-beating-cancer-plan-eu4health-financed-projects/projects/eu-canineq_en
PCM4EU (83) (Personalised Cancer Medicine for all EU citizens)	Combines the expertise of partners from 15 European countries to promote personalized cancer medicine	2021–2024	PCM4EU—European Commission. https://health.ec.europa.eu/non-communicable-diseases/cancer/europes-beating-cancer-plan-eu4health-financed-projects/projects/pcm4eu_en.
NewROAD (84) (Open Platform for European Networking and Repurposing of Oncological Assets and Drugs)	Focuses on developing an open, collaborative platform to repurpose drugs in oncology.	2021–2024	NEWROAD—European Commission. https://health.ec.europa.eu/non-communicable-diseases/cancer/europes-beating-cancer-plan-eu4health-financed-projects/projects/newroad_en.
I-VIOLIN (85) (Implementing verifiable oncological imaging by quality assurance and optimization)	Aims to satisfy the need to optimize and harmonize oncological imaging procedures in Europe.	2021–2024	I Violin—European Commission. https://health.ec.europa.eu/non-communicable-diseases/cancer/europes-beating-cancer-plan-eu4health-financed-projects/i-violin_en.c
CRANE (86) (Network of Comprehensive Cancer Centres)	Focuses on create a sustainable structure for the EU Network of National Comprehensive Cancer Centres.	2021–2024	V CraNE—European Commission. https://health.ec.europa.eu/non-communicable-diseases/cancer/europes-beating-cancer-plan-eu4health-financed-projects/projects/crane_en.
Interact Europe (87) (Innovative collaboration for Inter-specialty cancer training across Europe)	A project designed to foster new approach to inter-specialty cancer training.	2021–2024	INTERACT-EUROPE—European Commission. https://health.ec.europa.eu/non-communicable-diseases/cancer/europes-beating-cancer-plan-eu4health-financed-projects/projects/interact-europe_en.
JANE (88) (Joint Action on Networks of Expertise)	Focuses on enhancing the shaping seven EU Networks of Expertise for cancer care-related issues that require cross-border cooperation and European-level know-how. The issues include: complex cancers, poor prognosis cancers, palliative care, survivorship, personalized primary cancer prevention, ‘omics’ technologies, hi-tech medical resources and adolescents and young adults with cancer.	2021–2024	JANE—European Commission. https://health.ec.europa.eu/non-communicable-diseases/cancer/europes-beating-cancer-plan-eu4health-financed-projects/projects/jane_en.
BEACON (13)	The project aims to address cancer disparities, support decision-making and ensure sustainability.	2021–2024	Cancer Beacon—Beacon.
iBeChange (75)	The ultimate goal of the iBeChange project is to advance the objectives of Europe’s Beating Cancer Plan and the European Code Against Cancer by enhancing long-term primary cancer prevention through the information, support, and empowerment of EU citizens.	2023–2028	Projects—EAPM. https://euapm.eu/projects/.

Despite progress, significant inequalities persist throughout the entire cancer care pathway across the EU. For example, data from 2020 show that only Denmark and the Netherlands analyzed more than half of cancer biopsies using next-generation sequencing (NGS), while countries such as Czechia and Slovakia did not use NGS at all (2, 5). Access to immunotherapies and targeted therapies remains limited, with many countries continuing to rely on older chemotherapy regimens (6–8).

Between 2012 and 2020, the European Medicines Agency (EMA) authorized approximately 10 new cancer medicines annually. That number rose to 17 in 2021, and 15 in 2022 (9). However, access to newly approved anticancer drugs remains uneven across the EU. The time to reimbursement (TTR) for new medicines varies significantly, contributing to unequal access across both low-and high-income countries (10). Although the EU Transparency Directive sets a 180-day deadline for reimbursement decisions, studies show that the actual interval between EU marketing authorization (EU-MA) and national reimbursement decision differs substantially between countries (11, 12). According to the European Federation of Pharmaceutical Industries and Associations (EFPIA) and its Waiting to Access Innovative Therapies (W. A. I. T.) indicator, only 6% of recently approved medicines are accessible in Serbia, compared to 85% in Germany. Moreover, the time to access in Serbia is 15 times longer than in Germany, suggesting that national-level reimbursement delays are a key driver of these disparities (6).

This review highlights the importance of TTR for novel anticancer therapies and its role in perpetuating disparities in access across countries in EU. It examines how TTR and finally equally and timely access to innovative anticancer drugs is influenced by factors such as screening and early diagnosis, accessibility to specialized treatment centers, participation in clinical trials, and socioeconomic conditions. This analysis is framed within the BEACON project, an EU-funded initiative designed to identify and address cancer disparities in Europe (13). The project prioritizes the perspectives of four key stakeholders: primarily patients, followed by healthcare providers, researchers, and policymakers.

## Methods

This article is based on a narrative review of the literature addressing healthcare inequalities in cancer care across the European Union, with a focus on access to innovative therapies and time-to-reimbursement. A non-systematic search was conducted in PubMed, Scopus, and Web of Science, covering publications from database inception to September 2024. Search terms included: *cancer, health disparities, survival, new therapies, new treatment, time to reimbursement, Europe,* and *socioeconomic status.* Additional sources, such as official documents from the European Medicines Agency, the European Commission, and EU-funded project websites, were reviewed to supplement peer-reviewed literature. Priority was given to recent studies, European data, and publications reflecting multi-stakeholder perspectives. Articles were selected based on their relevance to the themes of reimbursement, innovation access, and regional or socioeconomic disparities in cancer care. This narrative review aimed to synthesize key themes, identify gaps, and contextualize them within the framework of the BEACON project.

## Approved therapies

The regulatory approval of anticancer drugs is primarily based on evidence of efficacy, safety, and a favorable benefit–risk profile. In recent years, the European Medicines Agency (EMA) has adopted a more flexible approval approach, reflecting the growing focus on personalized medicine and immuno-oncology. These efforts aim to shorten TTR across Europe and promote equitable access to innovative treatments for all European citizens (14).

To accelerate approval, a variety of regulatory tools have been introduced. Single-arm studies, particularly for rare cancers and tumor-agnostic indications, are increasingly accepted. In parallel, complex trial designs, such as umbrella, basket, and platform trials are gaining traction (15–18). In cases where therapeutic benefits are urgent and clearly demonstrated, regulatory decisions may be granted within 150 days, with post-authorization data collection mechanisms used to address remaining evidence gaps. Approvals under exceptional circumstances, often accompanied by specific safety requirements, and compassionate use programs can further expedite access (19, 20).

The PRIority MEdicines (PRIME) scheme supports accelerated assessments by fostering early dialogue between applicants and the EU regulatory network. Similarly, the ADAPT-SMART consortium explores managed entry agreements for adaptive pathway products, aiming to better align regulatory and market access processes. In Europe’s publicly funded healthcare systems, the success of adaptive pathways depends on close collaboration between manufacturers, payers, and health technology assessment (HTA) bodies, particularly during pricing and reimbursement negotiations (21, 22). The Medicines Adaptive Pathways to Patients (MAPPs) initiative seeks to balance “evidence and access,” enabling early availability of therapies for appropriate patient groups in a sustainable manner (22).

Although there is no universally accepted definition of drug value, pricing is a critical factor in the approval of new anticancer agents. The expanding pipeline of innovative drugs places increasing pressure on national and regional healthcare budgets, highlighting the urgency of developing sustainable pricing models. EMA pilot programs like MAPPs aim to align the interests of all stakeholders in this process. Additionally, tools such as the ESMO Magnitude of Clinical Benefit Scale (ESMO-MCBS) have been developed to better assess the clinical value of new therapies, distinguishing those that offer substantial therapeutic benefits from those that provide only marginal gain or potential harm (23, 24).

Importantly EMA regulatory approvals do not consider economic factors, as this responsibility lies with national authorities. Consequently, decisions about cost-effectiveness and efficiency are made at the national level, where economic considerations are integrated into health policy (25).

While there is no single, straightforward solution, access to innovative treatments can be improved through closer dialogue between key stakeholders. Delays in TTR are often driven by complex negotiations processes.

## Socioeconomic factors

Differences in cancer mortality between Western Europe (WE) and Central-Eastern Europe (CEE) are largely attributed to lifestyle related risk factors such as smoking and alcohol consumption (26). A study from the LIFEPATH project highlights that cancer mortality across Europe is predominantly driven by higher death rates among lower-education groups, underscoring the impact of socioeconomic inequalities (27). Ensuring early access to anticancer therapies across all socioeconomic groups is essential to addressing disparities and improving survival outcomes (28–30).

Several socioeconomic factors influence TTR for new oncology treatments. Two of the most critical are the presence or absence of formal assessment procedure prior to reimbursement decisions and a country’s gross domestic product (GDP) (30). Although CEE countries spend a comparable percentage of GDP on oncology drugs as WE and thereby prioritize cancer care, their absolute expenditure per capita and per cancer case is significantly lower (31). Moreover, Eastern and South-Eastern EU countries allocate five to six times less funding to cancer care compared to wealthier Western nations, resulting in markedly reduced access to innovative anticancer therapies (32–35).

In high-income countries, access to new anticancer drugs, is often facilitated by mechanisms such as the European Society for Medical Oncology’s Magnitude of Clinical Benefit Scale (ESMO-MCBS v1.1), the orphan drug scale, conditional marketing authorization (CMA), and accelerated approvals (AA). Access in these settings tends to correlate with higher GDP, streamlined reimbursement pathways, and active engagement by large pharmaceutical companies (10). Although innovation in oncology challenges even well-resourced health systems, its impact is more acute in lower-income regions where regulatory and reimbursement processes are slower and less consistent.

This disparity is evident in the time between marketing authorization and coverage application across EU countries. Denmark and Norway report some of the shortest intervals, followed by Belgium (15 days after EMA authorization) and Germany (20 days). Conversely, countries like Latvia (528 days), Greece (530 days), and Cyprus (716 days) experience significantly prolonged delays, reflecting systemic barriers to equitable access across Europe (36).

These socioeconomic discrepancies clearly reflect a threshold for accessing oncology drugs, highlighting the need for increased investment and more efficient, value-based use of available resources. Currently, EU investment in oncology drugs represents approximately 11% of total cancer-related spending, a relatively low figure, especially considering the role of new treatments in improving survival and reducing costs associated with morbidity and mortality (37, 38).

## Clinical trials

An additional strategy to reduce TTR and improve access to novel treatments is increasing enrollment in clinical trials. However, access to clinical trials remains unequal across the EU, with significant disparities affecting oncologists’ practices, particularly in Eastern and Southeastern EU countries (14). For example, lung cancer trials are primarily concentrated in Western Europe, limiting access for patients in other regions. A 2022 survey by Lung Cancer Europe revealed that 48% of lung cancer patients felt inadequately informed about available clinical trials (2, 39).

Improving access to clinical trials requires better dissemination of information among key stakeholders, including patients, researchers, and healthcare providers. This could not only accelerate patient access to new therapies but also facilitate earlier engagement with decision-makers. Legislative support for cross-border participation in clinical trials is essential for expanding access to promising treatments across the EU (14).

For patients, a key priority is access to user-friendly clinical trial search tools. Many find existing platforms, such as clinicaltrials.gov and clinicaltrialsregister.eu/, difficult to navigate (33, 40). Enhancing multilingual accessibility and offering training in clinical trial terminology could significantly improve understanding and engagement particularly among underserved or socioeconomically disadvantaged populations.

Clinicians also face difficulties in patient recruitment due to the large number of concurrent trials, which can fragment enrollment and limit the sample size available for individual studies. These constraints, combined with economic pressures to accelerate market access, often reduce the ability of individual trials to assess endpoints like overall survival and quality of life (QoL) (41). Addressing age-related disparities in inclusion criteria is also essential. Despite ongoing efforts, older adults are still underrepresented in oncology trials. Additionally, eligibility requirements that restrict participation to individuals aged 18 and over, often exclude adolescents and young adults from accessing innovative treatments (28).

The increasing use of biomarker-driven inclusion criteria, such as NGS, further complicates access to clinical trials. In countries where NGS tests are not reimbursed, participation in clinical trials may be the only available pathway for patients to access biomarker testing (42). Additionally, patients with rare oncological diseases often rely on clinical trials as their primary opportunity to receive experimental therapies, underscoring the need for transparent communication and equitable outreach strategies (43).

Improving access to information and addressing socioeconomic disparities across the EU are crucial for ensuring fair and inclusive participation in clinical trials and achieving equitable access to novel cancer therapies.

## Patient-reported outcomes and real-world evidence

To support evidence-based decision-making, both randomized clinical trial (RCT) data and real-world evidence (RWE) are essential. Together, they help bridge the gap between regulatory approval and national-level reimbursement by ensuring that clinical value is assessed within both scientific and health system contexts. This approach can ultimately help reduce TTR (44–46).

Although RCTs remain the gold standard for establishing efficacy and safety, they often do not reflect the complexity of routine oncology practice. Populations such as older adults, patients with comorbidities, and those under the age of 18 are typically underrepresented, despite forming a large portion of the real-world cancer population. Important factors like dose modifications, treatment sequences, and coexisting conditions—common in clinical settings—are often excluded from RCT protocols, though they significantly influence treatment outcomes (16, 47).

When used alongside patient-reported outcome measures (PROMs), real-world data (RWD) offers a more comprehensive picture of therapeutic performance, capturing both clinical effectiveness and quality of life. These insights are increasingly important for supporting value-based access frameworks and enabling equitable and timely reimbursement decisions (48, 49).

However, the lack of standardized and comprehensive data across Europe remains a major challenge. Many EU countries do not systematically collect or publish RWD on cancer treatment patterns or survival. Only 15 of 31 European countries report five-year cancer survival rates by type and overall; three report by type only, and 13 provide no such data at a national level. Moreover, most lack detailed, nationwide insights into treatment practices across hospitals and regions. These data gaps hinder the evaluation of real-world therapeutic value and contribute to delays in TTR (7, 50).

The European Medicines Agency (EMA) is increasingly considering RWE in regulatory assessments, especially via adaptive pathways. Still, its use remains mainly supportive. A review of EMA oncology approvals from 2018 to 2022 showed that RWE appeared in just 32% of European Public Assessment Reports (EPARs), and typically only as complementary evidence (10, 47).

To enhance RWE’s utility, groups like the European Organisation for Research and Treatment of Cancer (EORTC) are promoting pragmatic trial designs and cohort-based studies. Rapid-learning health systems, such as ASCO’s CancerLinQ, exemplify how real-time clinical data can support evidence-informed decisions (51).

Additionally, several EU countries are piloting outcome-based managed entry agreements, linking reimbursement to actual treatment outcomes—demonstrating RWE’s potential to improve transparency, accountability, and cost-effectiveness (52, 53).

In conclusion, integrating PROMs with patient-level RWD via national cancer registries could serve as the gold standard for monitoring treatment quality, supporting value-based pricing, enabling more objective TTR timelines, and reducing inequalities in access to innovative therapies across Europe.

## Expertise centers

Integrating innovations into clinical practice is essential for improving patient outcomes and reducing healthcare disparities. However, when access to advanced services is limited to university hospitals and comprehensive cancer centers, patients in rural or underserved areas are often excluded. As timely access to high-value treatments is a key factor in reducing TTR and expanding equitable cancer care, it is critical to ensure that innovative, evidence-based therapies are available across all regions.

Healthcare systems in Eastern and South-eastern Europe often face structural limitations, including insufficient capacity and a lack of specialized services, In contrast, Western European countries like Austria, Hungary, and Germany have established more comprehensive networks of expertise, including regional specialty centers and nationally certified cancer institutions (36). For example Italy experiences significant disparities, with specialized centers and multidisciplinary teams more commonly found in the northern regions than in the south (54).

To address these disparities and improve cancer care quality across Europe, the European Commission is advancing the creation of an EU Network of Comprehensive Cancer Centres (55). Multidisciplinary tumor boards (MDTs)—composed of oncologists, surgeons, radiologists, and pathologists—play a pivotal role in delivering high-quality, coordinated cancer care. The proposed network aims to standardize access to quality-assured diagnostics and evidence-based treatments, regardless of geographic location, while also fostering collaboration in professional training, research, and clinical trials throughout the EU (56, 57).

## Screening and early diagnosis

Screening programs and early diagnoses are fundamental to the effective treatment of early-stage diseases, as they improve patient outcomes and reduce cancer mortality-to-incidence ratios (58). They also offer the opportunity to optimize TTR by enabling earlier access to innovative therapies with higher curative potential.

Despite their clear benefits, early detection programs in Central and Eastern European countries often remain suboptimal, resulting in delayed diagnoses and worse outcomes. Currently, cancer screening participation rates in at least one-third of EU member states fall below 50%. Barriers such as limited educational attainment and geographic disparities continue to hinder participation in these programs (36, 59).

Conversely, recent advancements in screening technologies—particularly those involving artificial intelligence (AI) and genomics—are revolutionizing early cancer detection (60, 61). These innovations promise to enhance both diagnostic accuracy and efficiency. However, they also raise a pressing question: How does the effectiveness of early diagnosis impact the overall cost of new cancer therapies? This highlights the complex interplay between diagnostic innovation, treatment efficacy, healthcare expenditures, and TTR (62).

Focusing treatment on patients who are most likely to benefit, while minimizing overtreatment, represents a critical strategy for validating treatment programs and controlling pharmaceutical costs. This approach relies on the identification of predictive biomarkers and the application of precision treatment dosing to optimize outcomes, reduce unnecessary drug use and potentially shortening the TTR.

## Discussion

The TTR for innovative anticancer medicines varies significantly across EU member states and is often excessively long, limiting timely access to new treatments. Addressing this challenge requires improvements not only in drug development but also in the efficiency of national healthcare systems. Multiple key organizations contribute to the advancement and equitable access to novel therapies across Europe (Table 2). To ensure patients receive effective treatments promptly and affordably, greater collaboration is needed among regulators, payers, governments, patient advocates, and the pharmaceutical industry. However, there is no single, straightforward solution.

**Table 2:** This table provides several key organizations involved in the development and access to novel cancer medicines in Europe.

Overview of key organizations contributing to access and development of novel cancer medicines in Europe			
Organization	Country/Region	Role	Key contribution
European Medicines Agency (EMA) (89)	European Union	Regulatory Authority	Evaluates and approves new cancer medicines for the EU market.
European Commission (90)	European Union	Policy-Making Body	Develops health policies affecting access to medicines.
EUnetHTA (91)	European Union	Health Technology Assessment Network	Assesses clinical and economic value of new treatments.
National HTA Agencies, e.g., National Institute for Health and Care Excellence (NICE), Institute for Quality and Efficiency in Health Care (IQWiG) (92)	Various EU countries	National Agencies	Conduct HTA to inform reimbursement decisions.
European Society for Medical Oncology (ESMO) (93)	Europe-wide	Professional Society	Advocates for quality cancer care and education on treatments.
European Cancer Organisation (ECO) (94)	Europe-wide	Advocacy Organization	Focuses on policies to improve cancer care and access.
European Cancer Patient Coalition (ECPC) (95)	Europe-wide	Patient Advocacy Group	Represents cancer patients’ interests and advocates for access.
European Network for Cancer Research in Children and Adolescents (ENCCA) (96)	Europe-wide	Research Network	Promotes access to innovative treatments for childhood cancers.
Innovative Medicines Initiative (IMI) (97)	Europe-wide	Public-Private Partnership	Funds projects to accelerate the development of new medicines.
Cancer Core Europe (98)	Europe-wide	Collaborative Research Network	Enhances development of novel cancer treatments.
European Reference Network for Rare Adult Cancers (EURACAN) (99)	Europe-wide	Rare Cancer Network	Improves access to treatments for rare adult cancers.
European Cooperation in Science and Technology (COST) (100)	Europe-wide	Networking Program	Supports collaborative cancer research initiatives.
European Alliance for Personalised Medicine (EAPM) (75)	Europe-wide	Advocacy Group	Promotes personalized medicine approaches for cancer treatments.
European Organisation for Research and Treatment of Cancer (EORTC) (101)	Europe-wide	Clinical Research Organization	Conducts clinical trials for novel cancer treatments.

In response to these disparities, the Beacon project (BEACON) was established through a partnership involving the European Health and Digital Executive Agency (HADEA), Istituto Europeo di Oncologia (IEO), the European Alliance for Personalised Medicine (EAPM), Sporedata OU, Klinicki Bolnicki Centar Sestre Milosrdnice (SMUHC), and the University of Palermo (UNIPA) (13). BEACON aims to build a cross-border network that supports the uptake of quality-assured diagnosis and treatment, while also promoting training, research, and clinical trials across the EU. The project leverages digital technology to improve health literacy, with a focus on disadvantaged groups such as older adults, individuals from lower socioeconomic backgrounds, and populations in geographically disadvantaged regions (63, 64).

The project involves four stakeholder groups: patients, healthcare providers, researchers, and policymakers, and promotes dialogue across key issues, including access to innovative therapies, clinical trial participation, specialized care, and equitable screening, regardless of socioeconomic status (65).

**Figure 1:**
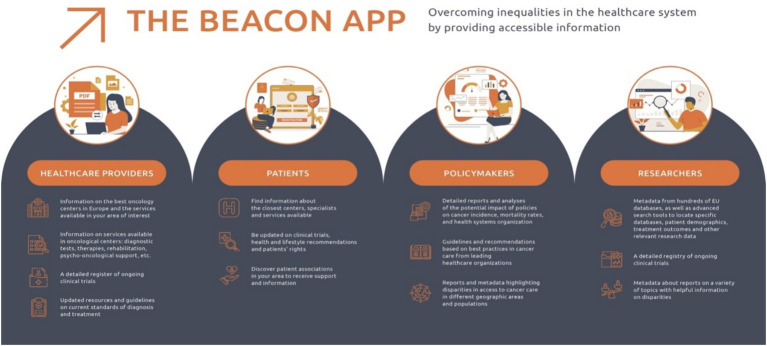
Stakeholder-centered functionalities of the BEACON App. The BEACON app offers tailored features for healthcare providers, patients, policymakers, and researchers to improve access to cancer care and reduce disparities across the European Union.

A key deliverable is the BEACON Decision Support System (Figure 1), a digital application based on a wiki platform, that enables the exchange of information among stakeholders. It bridges the gap between regulatory approval and reimbursement by integrating real-world evidence (RWE) and quality of life (QoL) data, considered equally important to traditional endpoints like overall survival (OS) and progression-free survival (PFS) (66). The platform also supports clinicians in making evidence-based treatment decisions, researchers in accessing the tools and resources needed to advance cancer research, and policymakers in gaining data-driven insights.

Most importantly, the app is designed to provide accessible, multilingual health information to patients, especially those in underserved or rural populations who often face barriers to healthcare. By systematizing complex medical concepts and offering clear guidance on clinical trials and innovative treatments, it aims to broaden access across diverse populations and eventually include patients with rare cancers (40, 67). In synergy with initiatives like the CAN.HEAL project, which aims to create a European public health genomics platform, the BEACON app promotes equitable access to precision diagnostics and healthcare (68, 69). Ultimately, the platform fosters alignment between clinical research and real-world implementation, while addressing operational challenges within multidisciplinary teams across the EU (70).

However, several potential limitations were acknowledged. First, scalability across EU member states has been constrained by resource availability, varying levels of digital literacy, health system maturity, and Information Technology (IT) infrastructure. Second, the long-term sustainability of BEACON has depended on continuous data updates and secured funding beyond the EU4Health grant period. To address this, a data curation system and a business exploitation plan have been developed to ensure continuity and relevance.

Implementation has followed an agile, participatory model using stakeholder interviews to co-design the interface. Based on the insights collected from the interviews, we will refine the use case to further explore stakeholders’ needs and preferences regarding the Beacon Decision Support System and the specific modalities in which information can be used within our project. The pilot testing will assess usability and engagement, with outcomes measured through qualitative and quantitative indicators, including user satisfaction, accuracy of shared information, and reported impact on care navigation.

**Figure 2:**
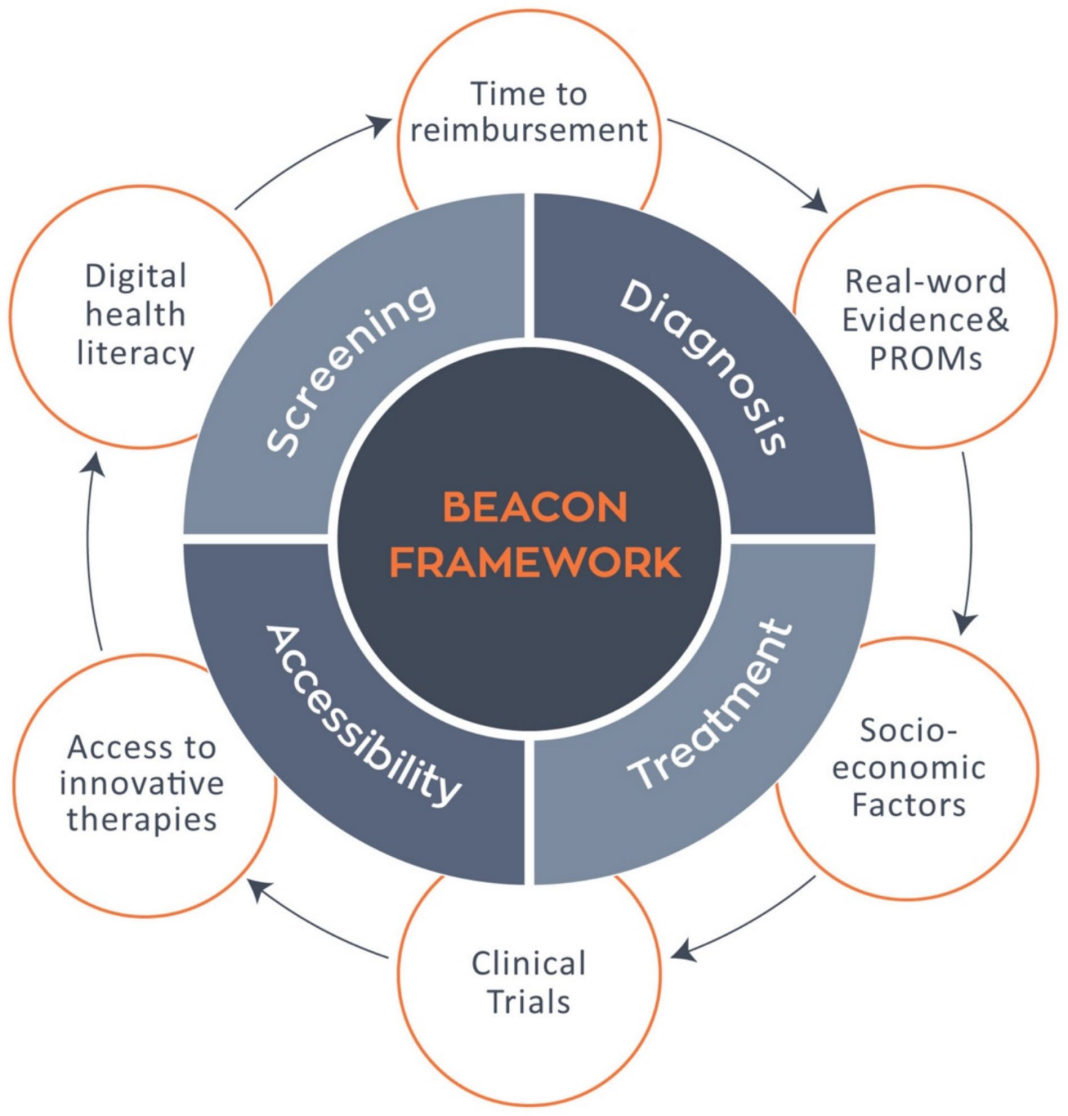
The BEACON framework within the cancer care continuum. The BEACON framework integrates key phases of cancer care screening, diagnosis, treatment, and accessibility with systemic determinants such as time to reimbursement, real word evidence and PROMs, socioeconomic factors, clinical trials, access to innovative therapies, and digital health literacy, aiming to reduce disparities and enhance equity across the European oncology landscape.

The BEACON project thus delivers both theoretical and practical value (Figure 2). Conceptually, it promotes a model that integrates health outcomes with structural determinants to reduce TTR. Practically, it offers scalable tools and frameworks aligned with EU metrics, enabling real-world application across diverse healthcare settings.

## Conclusion

Nobel laureate Joseph Stiglitz powerfully stated, “*Inequality is a choice, not a destiny*” (71). This statement underscores that health disparities are not inevitable, but shaped by policy decisions and institutional priorities. Ensuring equal access to innovative therapies is essential for achieving equity across Europe.

To improve the TTR process for novel drugs, procedures must be streamlined to enhance communication and coordination across the European Union’s healthcare system. The BEACON initiative reflects this aim by fostering collaboration among key stakeholders, empowering patients through accessible information, and promoting data driven, value based decision making.

In particular, the BEACON app is positioned to become a vital tool in bridging the gap between innovation and implementation, ensuring that novel cancer therapies reach patients regardless of geography or socioeconomic status.
